# Efficacy of cycled environmental light and noise during initial hospitalisation for improved cognitive outcomes at 2 years in infants born extremely or very preterm: study protocol for the prospective, randomised, open, blinded endpoint controlled multicentre CIRCA DIEM trial

**DOI:** 10.1136/bmjopen-2025-112665

**Published:** 2026-07-24

**Authors:** J Jane Pillow, Rod W Hunt, Julie A Marsh, Peter J Anderson, Peter J Mark, Alicia J Spittle, Andrew J O Whitehouse, Nadia Badawi, The CIRCA DIEM Study, Natasha Sorensen

**Affiliations:** 1 School of Human Sciences, The University of Western Australia, Perth, Western Australia, Australia; 2 Developmental Chronobiology, Strong Beginnings, The Kids Research Institute Australia, Perth, Western Australia, Australia; 3 Paediatrics, Monash University, Clayton, Victoria, Australia; 4 Cerebral Palsy Alliance Research Institute, The University of Sydney, Camperdown, New South Wales, Australia; 5 The University of Western Australia, Perth, Western Australia, Australia; 6 The Kids Research Institute Australia, Perth, Western Australia, Australia; 7 Department of Pediatrics, University of California Irvine, Irvine, California, USA; 8 Physiotherapy, University of Melbourne, Parkville, Victoria, Australia; 9 Victorian Infant Brain Studies, Murdoch Childrens Research Institute, Parkville, Victoria, Australia; 10 Grace Centre for Newborn Intensive Care, The Sydney Children’s Hospitals Network, Westmead, New South Wales, Australia

**Keywords:** Infant, Cognition, Clinical Protocols, Intensive Care Units, Neonatal, NEONATOLOGY

## Abstract

**Introduction:**

Very preterm infants (<32 weeks gestation) have an undeveloped primary endogenous circadian rhythm and are deprived abruptly of vital maternal circadian inputs after delivery. Postnatal care in neonatal intensive care units is characterised by constant levels of lighting and noise. Stress arising from disrupted cues for circadian rhythmicity likely disrupts development of coordinated circadian rhythms critical for neurogenesis, organ growth and development. We hypothesise that cycled environmental light and noise commenced soon after birth and continued until discharge home will improve cognitive outcomes compared with infants whose postnatal care comprises constant light (bright or dim) and constant noise.

**Methods and analysis:**

Australasian multicentre, two-arm, parallel-group, prospective, randomised, open, blinded-endpoint superiority trial in 868 infants born less than 32 weeks’ gestation. Infants are randomised to cycled environmental light and noise or routine care in a non-cycled hospital environment from soon after birth until discharge home. The intervention comprises wearing eye-masks and ear plugs from 20:00 to 6:00, followed by removal of these devices and exposure to normal environmental noise and 300-600 lux light from 6:00 to 20:00. The primary outcome is composite cognitive score on Bayley-4 developmental assessment at 2 years corrected postnatal age.

**Ethics and dissemination:**

The trial is approved by the Child and Adolescent Health Service Human Research Ethics Committee under the National Mutual Acceptance Scheme in Australia. Infants are randomised to intervention or control group after informed parental consent is obtained. Results of the CIRCA DIEM Study will be disseminated widely via presentations at local, national and international conferences, publication in international peer-reviewed journals and inclusion on the study website. Information about trial findings will also be communicated directly to the parents/guardians of trial participants through the regular study newsletter. The trial investigators will seek opportunities to communicate study results to the lay public through media and social media avenues.

**Trial registration number:**

ANZCTRN12618000371291.

**STRENGTHS AND LIMITATIONS OF THIS STUDY**

- The study is powered to identify a 4-point increase in cognitive scale on the Bayley-4 assessment at 2 years corrected postnatal age.
- The study will use a rigorous statistical analysis using the Estimands Framework.
- The study protocol incorporates an integrated biobank and physiological substudies to facilitate future discovery research to explore mechanisms underlying clinical outcomes.
- While inability to blind the intervention is a study limitation, the trial steering committee, all outcome assessors and study statisticians are blinded to group assignment.

## Introduction

### Background and rationale

#### Burden of illness

 Preterm infants are at increased risk of chronic health problems including neurodevelopmental conditions, cerebral palsy, mental health, respiratory, renal, cardiovascular, metabolic, infectious and cancerous complications in later life,^1 2^ each of which is associated with chronodisruption.^3^

Cognitive impairment is the major and most distressing disability arising from preterm birth and the primary concern for parents and carers of preterm infants after hospital discharge. Premature babies also exhibit higher rates of adverse motor, behavioural and psychiatric outcomes in late childhood compared with children born at term gestation, even in the absence of recognised perinatal brain injury.^4^

#### Role of circadian rhythms in development

The vital role of an intact circadian rhythm for development of multiple body organs and systems is increasingly evident, and especially for the developing brain. The fetus is dependent on maternal circadian physiological (eg, temperature; melatonin and cortisol), auditory, tactile and kinaesthetic sensory cues to orchestrate developmental processes until near term.^5 6^ These maternal cues entrain the fetal circadian rhythms to the external light-dark cycle prior to development of endogenous fetal circadian rhythms.^7^

#### Effect of preterm birth on development of circadian rhythms in the premature newborn

Very preterm infants (<32 weeks gestation) have an undeveloped primary endogenous circadian rhythm^8^ and are deprived abruptly of vital maternal circadian inputs after delivery. Furthermore, very preterm infants are cared for in neonatal intensive care units (NICUs) characterised by constant levels of lighting and noise and are exposed to neuroactive medications (eg, caffeine, glucocorticoids) at non-physiological times. Stress arising from disrupted cues for circadian rhythmicity likely disrupts development of coordinated circadian rhythms critical for neurogenesis, organ growth and development,^9 10^ including disruption of white matter organisation,^11^ a critical determinant of cognitive function in childhood.^12^ The lack of an endogenous circadian rhythm for infants born preterm may also detract from the quality and quantity of sleep,^13^ which is critical to neurodevelopment.^14^ Disrupted circadian rhythms also reduce capacity for selective attention and executive function^15^ that are frequently impaired in survivors of preterm birth,^16 17^ and increase vulnerability to autism spectrum disorder. Importantly, circadian disruption also exerts key programming effects on the developing clock, increasing risk for later metabolic disease.^18 19^ Infants born very and extremely preterm are at highest risk of prolonged chronodisruption, due to the extended period of hospitalisation prior to discharge to a home environment.

#### Current recommendations about environmental conditions in the NICU

The American Academy of Pediatrics (AAP) suggested introducing regular cycles of day-night lighting in the NICU in 1997.^20^ However, there is no substantive clinical evidence to support this practice and few units achieve adequate (200 lux) separation of artificial night and day. To the contrary, the widely implemented Neonatal Individualised Developmental Care and Assessment Program recommends continuous dim lighting (<20 lux) for 24 hours/day during early postnatal life, to emulate the darkened environment of the uterus.^21^ However, this continuous dim lighting eliminates any environmental zeitgeber input to the suprachiasmatic nucleus (the ‘master clock’), which the fetus normally receives from circadian maternal cues. Importantly, even low levels of white light inhibit melatonin secretion, and in animal studies result in increased anxiety and delayed growth.^22^ The most recent Cochrane systematic review of cycled light in the intensive care unit for preterm or very low birth weight highlighted the need for a large, multicentre study with long-term follow-up. Most studies performed to date have evident or uncertain selection bias, and lack of clarity around outcome assessment (detection bias) and reporting bias, and involved single centres. The impact of cycled light for neurodevelopmental outcomes has not been considered. Importantly, preterm infants who are exposed to cycled light from birth develop circadian rhythmicity in melatonin profiles as early as 5 days of age.^23 24^ This observation indicates they can respond to cycled light and is consistent with the timing of functionality in the retinohypothalamic pathway.^25^

The AAP recommends that NICU noise levels should not exceed 45 dB,^18^ but this maximum exposure is rarely achieved with NICU noise levels often between 55 and 70 dB.^26^ There are no current recommendations about cycled noise in the NICU and it is unknown whether noise is a zeitgeber that entrains circadian rhythms. There are no studies of cycled noise exposure in preterm infants. However, one small (n=24) study evaluated the use of silicone earplugs worn throughout the admission in very low birth weight (<1500 g) infants within a single centre. A mean (95% CI) increase in weight at 34-week post-menstrual age (PMA) of 225 (45, 405) g was noted in the group assigned to earplugs compared with controls. Follow-up at 18–22 months was only completed for 12 infants, but reported mean (95% CI) increases in the Bayley Mental Developmental Index of 15.5 (3.0, 28.0) points and in head circumference of 2.6 (1.0, 4.2) cm for the six infants that wore the earplugs compared with the six infants in the control group.^27^ These promising outcomes need to be validated in an adequately powered trial with a primary neurodevelopmental outcome.

### Choice of comparators

Numerous environmental cues in hospital nurseries can delay development of endogenous rhythms, including light, noise, temperature regulation, feeding schedule and timing of neuroactive medications such as caffeine and steroids. Of these, light, noise and medication timing are likely the major disruptors of endogenous rhythms and are also most easily modified. The most recent Cochrane systematic review of cycled light for preterm infants acknowledged that the effect of a combination of interventions may be important for postnatal development of circadian rhythms in hospitalised preterm infants, rather than cycled light alone.^28^

#### Significance

Cycled environmental care is based on solid physiological principles and the increasing awareness that long-term well-being across multiple health domains is critically dependent on the integrity of circadian functions.^1^ It provides a simple, low-cost, attractive and pragmatic option for early postnatal development of endogenous circadian rhythms. The incorporation of these principles into the pragmatic design of the multicentre CIRCA DIEM trial will facilitate broad generalisability. A positive trial outcome would enable immediate adoption of cycled environmental care as routine neonatal management, with a potential to have lifelong health, welfare and socioeconomic benefits for the infants and their families.

### Objectives

#### Aim

To establish whether targeted individual diurnal cycling of environmental light and noise levels results in improved neurodevelopmental social, psychological, physiological and economic outcomes of infants born very preterm compared with more constant background lighting and noise environments.

#### Hypotheses

##### Primary hypothesis

Exposure to cycled light and noise from birth until discharge home will increase median cognitive score on the Bayley-4 at 2–3 years corrected postnatal age (cPNA) by 4 points compared with care in more constant background noise and lighting environments.^29^

##### Secondary hypotheses

Compared with more constant background noise and lighting environments, exposure to cycled light and noise from birth until discharge home will:

1. improve neurodevelopmental scores at 2 years cPNA;
2. not increase mortality or neonatal morbidities;
3. result in more rapid development of endogenous circadian rhythms;
4. reduce parental depression and anxiety and improve parent–infant interactions and
5. decrease socioeconomic costs of premature birth.

## Methods

### Patient and public involvement

A consumer representative (Catherine Gwynn) and the Preterm Community Reference Group at The Kids Research Institute Australia will provide consumer and community input to the steering committee, trial design and trial materials including reporting of outcomes to incorporate patient and family perspectives.

### Trial design

**Figure 1:**
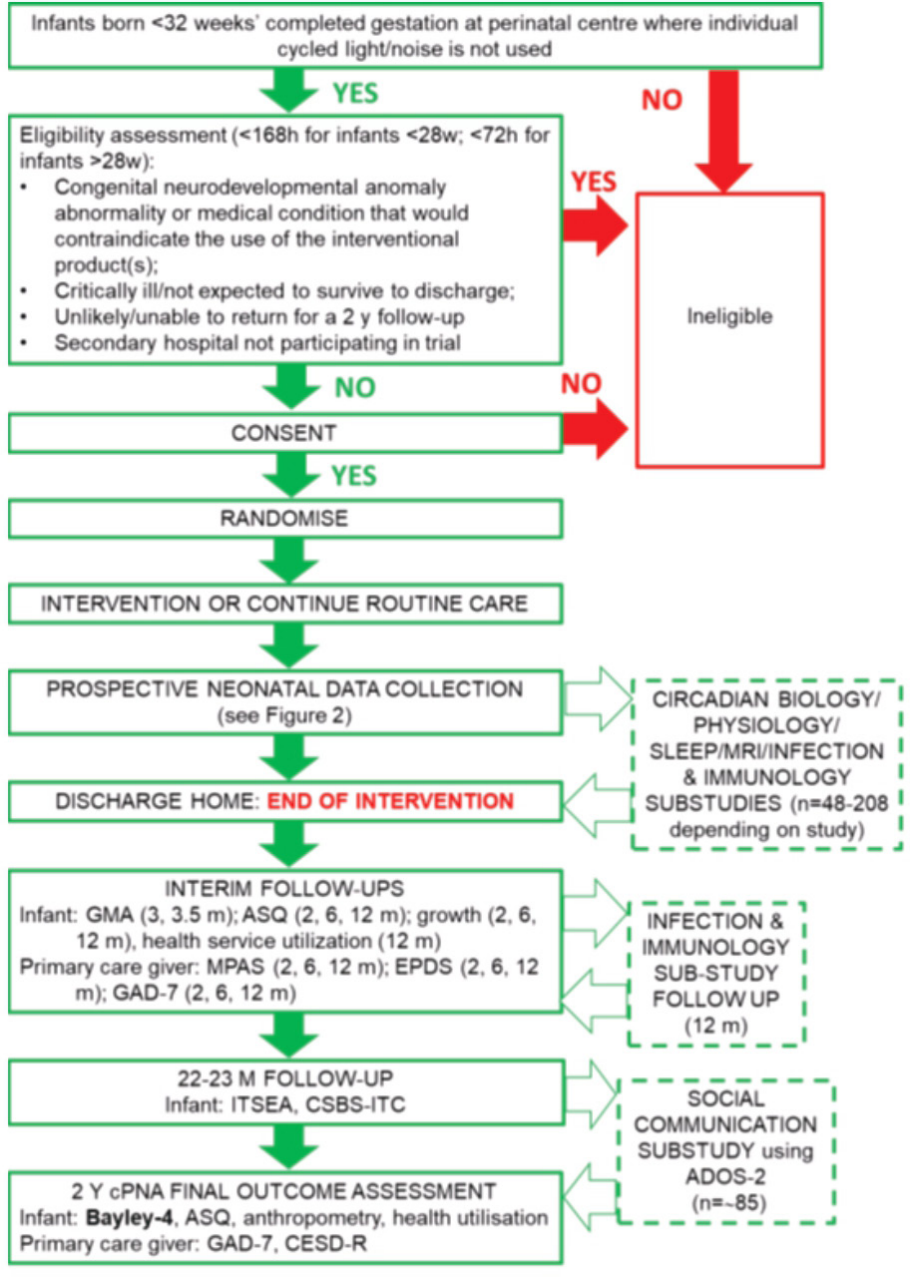
CIRCA DIEM Study flow chart. ADOS-2, Autism Diagnostic Observation Schedule, Second Edition (Toddler Module); ASQ, Ages and Stages Questionnaire; CESD-R, Centre for Epidemiologic Studies Depression Scale–Revised; cPNA, corrected postnatal age; CSBC-ITC, Communication and Symbolic Behaviour Scales Infant-Toddler Checklist; EPDS, Edinburgh Postnatal Depression Scale; GAD-7, Generalised Anxiety Disorder Scale-7; GMA, General Movements Assessment; ITSEA, Infant Toddler Social Emotional Assessment; MPAS, Maternal Postnatal Attachment Scale.

The CIRCA DIEM is an Australasian multicentre (11 sites), two-arm, parallel-group, prospective, randomised, open, blinded-endpoint (PROBE) superiority trial undertaken in a broad group of infants at highest risk for prolonged chronodisruption (very and extremely preterm infants) with a primary neurodevelopmental outcome (cognition). The participant flow chart for the study is shown in figure 1.

### Study setting

The study will be undertaken in NICUs in major (tertiary) perinatal centres (primary recruiting sites) and supporting regional units (secondary sites) across Australia and New Zealand. The trial commenced on 4 February 2019. Last data collection is anticipated by 30 June 2029.

### Eligibility criteria

A register of potentially eligible infants will be documented by each study site to ensure transparent reporting of the study population.

#### Inclusion criteria (all must be satisfied)

1. born at <32 weeks PMA by best obstetric estimate (inborn or outborn).
2. initial care is at a perinatal centre where routine care does not include formal environmental light/noise cycling.
3. informed parental consent to trial participation, obtained either prior to or after birth (<168 hours for infants <28 weeks PMA, or <72 hours for infants ≥28 weeks PMA and <32 weeks PMA).

#### Exclusion criteria (any one or more mandates exclusion)

1. have a congenital neurodevelopmental abnormality or medical condition that would contraindicate the use of the interventional product(s)
2. are critically ill and not expected to survive
3. unwilling or deemed unlikely to return for Bayley-4 follow-up at ~2 years cPNA; (eg, >200 km from tertiary centre and not enrolled in funded follow-up programme, itinerant family)
4. anticipated discharge to a non-participating neonatal unit before 8 weeks postnatal age (for infants <28 weeks PMA).

### Intervention and comparator

Continuous light (either continuous bright light or continuous dim light/near darkness) is current standard of care in most NICUs. Continuous dim light generally targets 5–20 but, in some units, may include background lighting up to 100 lux. Cot covers typically remain in place 24 hours/day in the first weeks/months after birth. Eye masks are routinely only used for brief periods in association with phototherapy treatment, when they are worn 24 hours/day.

Most neonatal units do not routinely use ear protection for cycled day/night exposure to environmental noise, although earmuffs are occasionally applied for 24 hours/day for short periods when infants are receiving high-frequency ventilation.

#### Intervention description

##### Light

For the intervention group, the CIRCA DIEM Study will target cycled light from 0 lux at night to a range of 300-600 lux during daytime as this level was defined as safe for preterm infants.^26^ We thus anticipate a minimum separation of 200 lux between the intervention group and comparator group for daytime light exposure,^30^ and a 300 lux difference between daytime/night-time light intensity for the intervention group to ensure adequate light stimulus for entrainment of the endogenous circadian rhythm. The intervention will commence as soon as possible after randomisation and continue until discharge home or (for infants hospitalised for longer than 8 weeks) for a minimum of 8 weeks after commencement of the intervention.

Conversely, the CIRCA DIEM Study will use a pragmatic definition of non-cycled light environment for the comparator group as a difference between daytime and evening light intensity that does not exceed 100 lux.

##### Noise

There is no information on what difference in daytime/night-time noise might promote circadian rhythm entrainment in animal or human data. For the intervention group, we therefore use a pragmatic approach with application of ear plugs overnight to reduce background and peak noise exposure of approximately 22 dB. The application of these earplugs will reduce noise exposure overnight for most infants in the intervention group to levels that approach or minimally exceed the maximum exposure of 45 dB recommended by the AAP.^31^

Conversely, the comparator group will not receive any hearing protection unless prescribed 24 hours/day for short periods for clinical reasons (eg, receiving high-frequency ventilation).

##### Application of the study intervention

Ear plugs and eye masks will be applied in the evening at 20:00 and removed at 6:00 the following morning to provide a 10-hour intervention night period that still allowed parent–infant eye contact and sound for evening parental visits. Cot covers may also be used overnight. Eye masks can be removed for breast feeds but will otherwise stay in place overnight to minimise risk of inadvertent exposure to white light during procedures. Portable bedside lux metres are used to adjust bedside lighting to ensure 300-600 lux lighting exposure is achieved in the daytime.

### Criteria for discontinuing or modifying allocated interventions

The intervention may be discontinued prior to discharge home in participants randomised or allocated to the intervention group if they (1) received a minimum of 8 weeks of the intervention and are being discharged to a non-participating regional unit or (2) have reached term equivalent (40 weeks PMA) and are being transferred to another hospital ward for long-term care. Discontinuation of the intervention, or modifying the intervention (eg, avoiding light exposure during the day during periods of critical illness) outside these criteria will represent a protocol deviation, including discontinuation of the intervention due to an adverse event attributed to the trial intervention as assessed by the local site investigator. Attending physicians may withdraw a participant from the study if they develop a study related complication. Parent(s)/guardians may withdraw their infant from the study at any time. Parental/guardian permission to continue to collect infant data after study withdrawal will be sought.

### Strategies to improve adherence to the intervention

Strategies to improve adherence to the intervention include (1) preinitiation site evaluation to ensure that the unit does not already cycle light and that it can achieve the light intensity levels specified within the protocol; (2) intensive site initiation and ongoing educational support programme from the trial co-ordinating centre and site research staff; (3) provision of portable bedside light lux metres and check logs to ensure that nursing staff are aware of lighting conditions and are able to adjust them to meet the specified daytime light intensity; (4) prespecified monitoring of light and noise levels of 24 hours on days 7, 14, 28 and 56 and at 36 weeks PMA; (5) regular monitoring of screening logs and data entry; (6) regular discussion about adherence and compliance issues and sharing of solutions and approaches at site investigator meetings and (7) additional online trial meetings and trial newsletters to sites and participants.

### Relevant concomitant care permitted or prohibited during the trial

Eyemasks may be applied for 24 hours/day in both the intervention (active comparator) and routine care (placebo comparator) group during any period when the infant is receiving phototherapy, in accordance with standards of care. Similarly, earmuffs may be worn for 24 hours/day in infants during high-frequency ventilation where that approach is standard care in that environment. Infants may still participate in the routine practice of afternoon ‘quiet time’ if required. This practice allows the dimming of light for 1–2 hours in the afternoon but does not permit the use of cot-covers, eye-masks or earplugs during this period.

Caffeine therapy will be timed in both study groups to be given with the first morning medications at or as close as possible to 8:00. Postnatal steroid administration, when required, will be timed so that the evening dose is given no later than 18:00 and the morning dose no earlier than 6:00. All other postrandomisation management of study infants is at the discretion of the clinical team.

### Outcomes

#### Primary outcome

Standardised cognitive score on Bayley Scales of Infant and Toddler Development–4th Edition at 24 (22–36) months cPNA.

#### Secondary outcomes

##### In hospital secondary outcomes

1. Survival to 36 weeks PMA.
2. Indices of growth: weight, length, head circumference, body mass index z scores and growth velocity (also measured at 2, 6 and 12 months cPNA).
3. Duration of hospitalisation to discharge from tertiary hospital and to discharge home.
4. Indicators of respiratory illness including
  - Incidence of bronchopulmonary dysplasia (BPD) (defined by Jensen *et al*).^32^
  - Severity of BPD (as defined by Jensen *et al* and right shift of the SpO_2_/P_I_O_2_ curve at 36 weeks PMA).^32 33^
  - Requirement for respiratory support as determined by duration of mechanical ventilation, duration of any respiratory support and days of supplemental oxygen.
5. Necrotising enterocolitis (NEC) Stage II or greater according to modified Bell’s Criteria.^34^
6. Severe retinopathy of prematurity (ROP) stage III or more, or ROP Stage II plus as defined in the International Classification of ROP 3.^35^
7. Neurologic injury as defined by
  - Grade 3 or 4 intraventricular haemorrhage (IVH).^36^
  - Presence of periventricular leukomalacia.
8. Number of confirmed blood culture positive episodes of sepsis.

##### Key long-term secondary outcomes at 24 (22–36) months cPNA

1. Survival, censored at 24 months cPNA.
2. Proportion of scores >1 SD and >2 SD below mean for cognitive composite score on Bayley-4.
3. Individual composite subscores for language, motor and social-emotional subscales of the Bayley-4.
4. Neurobehaviour using the Infant Toddler Social-Emotional Assessment.^37^
5. Social communication using the Communication and Symbolic Behaviour Scales Infant-Toddler Checklist (CSBS-ITC).^38^
6. Measures of growth: weight, height, body mass index, growth velocity.

##### Additional secondary outcomes to be reported in substudies

1. Risk of neuromotor disorder from assessments of the General Movements Assessment at 36 weeks PMA, and 3 m and 3.5 m cPNA.
2. Maternal–infant attachment using the Maternal Postnatal Attachment Scale at 2, 6 and 12 months cPNA.^39^
3. Primary caregiver mental well-being at 2, 6, 12 and 24 months cPNA
  - Anxiety score using the Generalised Anxiety Disorder-7.^40^
  - Depression using the Edinburgh Postnatal Depression Scale (2, 6 and 12 months)^41^ or the Centre for Epidemiologic Studies Depression Scale Revised at 24 months cPNA.^42^
4. Measures of sleep defined by actigraphy including total average time asleep/24 hours at 36 weeks PMA, and 2 months and 6 months cPNA for the infant and at 2 months and 6 months for the primary caregiver.
5. Time to onset of circadian rhythms measured by salivary melatonin and cortisol at day 7, 14, 28 and at 36 weeks PMA during hospitalisation.
6. Score on Toddler module of the Autism Diagnostic Observation Schedule (ADOS) Second Edition at 24 months cPNA.

##### Planning for long-term outcomes beyond the primary study endpoint

Long-term outcomes will include assessment of health service utilisation and medications where parents have provided us with consent to obtain this information.

### Cost-effectiveness analysis

Economic evaluation will ascertain the cost-effectiveness of cycledlight and noise compared with routine non-cycled environmental care over the first 2 years of life and will include estimation of long-term outcomes. Net savings will be estimated by comparing the length of stay with the costs of the intervention. The intervention cost, at a single primary recruiting site, will be assessed by prospective resource unit tracking including materials and time required for application/removal of eye-masks and noise protection. Resources will be costed using standard sources. Effectiveness over the longer-term will comprise evaluations of health-service utilisation.

### Adverse events

Adverse events during the intervention period will be assessed from randomisation until death or discharge home from hospital, or 44 weeks PMA, whichever occurs soonest.

The 2016 recommendations of the National Health and Medical Research Council for safety reporting^43^ will be followed by the CIRCA DIEM trial.

Study related adverse events during the intervention period will include

1. ROP with a stage III or greater, or stage II with Plus disease on International Classification of Reinopathy of Prematurity Third Edition.
2. Skin damage from use of wearables.
3. Any perceived safety issue associated with the intervention devices.

Study related serious adverse events during the intervention period will include

1. Death before discharge home from hospital deemed potentially associated with the intervention.
2. Any perceived life-threatening event during hospitalisation associated with the intervention devices.

Study-related serious adverse events occurring following the intervention period will include death after discharge and before 2 years of age associated with the intervention.

#### Reporting of adverse events and assessing their relatedness (causality) to trial interventions

The site principal investigator is responsible for reporting all predefined adverse events and serious adverse events from time of study enrolment to hospital discharge to the Trial Coordinating Centre, and grading the causality of these events as unrelated, possible, definite in relation to their causality with respect to the use of the study intervention. The aim will be that such reporting is achieved within 24 hours of the site investigator becoming aware of the event. All intervention-related SAE will be reviewed by the Data Safety and Monitoring Committee (DSMC) with the goal of providing a blinded report to the Chair of the Trial Steering Committee within 2 weeks of the review. The DSMC will additionally consider the composite outcome of mortality before discharge or severe IVH (grade 3 or 4 IVH), or NEC (stage IIA or higher) or ROP requiring treatment, in consideration of the safety of the intervention.

### Participant timeline

The flow chart for the study intervention is shown in figure 1. A schedule of enrolment, assessments and visits are shown in online supplemental table S1.

### Sample size

The initial sample size for the CIRCA DIEM study planned to recruit up to 954 babies to have over 90% power to detect a meaningful change in cognitive score of 4 points, which we defined as the lowest difference considered sufficient to promote a change in clinical care. A sample size re-estimation was planned at 75% completion of recruitment.

Sample size is based on a median difference of 4 points in the primary outcome (standardised cognitive score on the Bayley-4). The standardised score for cognition on Bayley-4 in the general population is designed to have a mean of 100 with an SD of 15.^29^ However, asymmetric distributions may be observed in the trial population. A planned sample size re-estimation was performed after 75% participants had been recruited, using simulation in the sim.ssize.wilcox.test function from the MKpower package.^44^ With the revised sample size of 624 (312 in each group), the study has 90% power to detect an absolute median difference in standardised cognitive score on Bayley-4 of at least 4 points, assuming a score of 85 (corresponding to –1 SD) and SD of 15 in the control group. The CIRCA DIEM study will recruit at least 868 infants (434 in each group) to allow for an anticipated 10% loss to follow-up (LTFU), and an incidence of multiple birth of 20%.

### Recruitment

Centres with a positive research culture, capacity to achieve the trial intervention and a clearly stated position on equipoise for the research question are included as primary study sites. Co-recruitment to other studies conducted in parallel is permitted to address issues of feasibility of recruitment targets in this highly powered trial within an in-demand finite population of very preterm and extremely preterm infants.

### Randomisation

#### Sequence generation

Computer-generated randomisation tables were developed and uploaded by the study biostatistician (JAM) from The Kids Research Institute Australia (The Kids), Perth Australia. Treatment allocations were generated using random permuted block sizes, stratified by study centre and gestational age (<28 weeks’ PMA vs 28.0 to <32.0 weeks’ gestation), maintaining 1:1 randomisation of infants to each study group.

#### Allocation concealment mechanisms

Infants are randomised to intervention or standard care via the concealed randomisation facility built into a web-based study Research Electronic Data Capture (REDCap, Vanderbilt University) database.^45^ Individuals performing randomisation will be unable to access the randomisation sequence. The REDCap database uses a checklist to confirm eligibility prior to randomisation.

#### Implementation

Research staff can screen for infants and approach families for informed consent antenatally or postnatally in the first 7 days (168 hours) for infants in Strata 1 (<28 weeks’ PMA) or in the first 72 hours for infants in Strata 2 (28.0 to <32.0 weeks’ PMA). Infants are randomised to either intervention (cycled light and noise) or routine environmental care

For multiple births, the first infant will be randomised and second and subsequent siblings from the same pregnancy will be allocated to the same group as the randomised first sibling. This approach is used to avoid any confounding from conflicting sibling sleep schedules that may impact on the primary outcome if the intervention alters sleeping behaviours with an effect extending beyond the initial period of hospitalisation.

#### Blinding

No attempt is made to blind parents or clinicians to the allocation group due to the extended duration of the intervention, and inability to blind carers and parents to the allocated group. However, assessors of primary outcome, and data analysts will perform their assessment and analyses blinded to study group allocation. Parents will be instructed not to inform assessors of their infant’s study group when they attend for follow-up assessment. Data collection will be performed by research staff uninvolved in clinical care of the infants. Analyses will be performed by a statistician independent of the trial research staff and investigators. Interim results will only be shared with the DSMC.

### Data collection methods

#### Data collection methods

Study staff are trained in data entry by the clinical trial coordinator prior to commencing data entry. Outcome assessments use validated data instruments as described in section 16. The collected data will include (but not be limited to) the following screening and baseline data categories in addition to the outcomes described previously.

1. Eligibility and randomisation data.
2. Maternal demographic data.
3. Infant baseline demographic data.
4. Randomisation data.

The statistical analysis plan (SAP) for the main study and for each of the key secondary studies will detail the baseline and screening data to be included in the final trial report.

#### Plans to promote participant retention and complete follow-up

Strong links with trial participants and their families will be maintained through trial updates, birthday cards for trial participants and regular e-newsletters. Families will be informed about intent to continue multimodal assessments that target a range of potential outcomes that may be influenced by this perinatal intervention, and consent to contact them for potential involvement of their child in such future studies will be obtained.

### Data management

CIRCA DIEM study investigators at each site will be responsible for data collection from medical notes (mother and infant), parents, and bedside clinical charts. Data collection will primarily use a custom-built database in the REDCap designed and managed by the Trial Management Group.^45^ Paper case report forms (CRF) will be used for bedside collection of 24-hour monitoring data and consents as appropriate and digitised for storage. Electronic CRFs will be checked for completeness and accuracy by the research team against source data and verified by the Trial Management Group. All data will be securely stored for the minimum period as per local legislated requirements (normally 15 years for parental data, 25 years for infant participants). Data will be securely destroyed/deleted after mandatory retention periods expire unless consent has been obtained to retain the data for follow-up studies that extend into later adult life of infant participants.

### Statistical methods

#### Statistical methods for primary and secondary outcomes

A separate detailed SAP for the core CIRCA DIEM study outcomes will be produced by a blinded trial statistician and finalised prior to completion of the recruitment phase. Additional SAPs may be published ahead of database lock for the analyses of CIRCA DIEM substudies. Reported results will be in accordance with Consolidated Standards of Reporting Trials (CONSORT) guidelines. Estimands will be constructed for all outcomes and detailed in the SAP. In brief, continuous outcomes will be analysed using either the Wilcoxon-Mann-Whitney test or linear mixed effects regression, binary outcomes using logistic regression and time-to-event outcomes using proportional hazards models, adjusting for the covariates listed above. The false discovery rate employed for all secondary analyses will use the Benjamini-Hochberg algorithm.

After the final database lock, data will be analysed in R Statistical Programming software^44^ using the principle of intention to treat by the blinded trial biostatistician (JAM). Comparability of study groups at baseline will be based on summaries of participant demographic data. Descriptive statistics will include mean (SD), median (IQR) for continuous variables with symmetric and asymmetric distributions, respectively, and counts/percentages for categorical variables.

##### Primary analysis

Standardised cognitive scores will be compared between the intervention and control group at 24 (20–36) months cPNA (primary outcome) using the Wilcoxon-Mann-Whitney test and summarised using the median of differences (Hodges-Lehmann estimator), with asymptotic 95% CI. The primary estimand will be detailed in the SAP, excluding non-randomised siblings from multiple births. In brief, missing primary outcome data due to death will be replaced with a score of 55 (corresponding to –3 SD), or with a score of 40 (–4 SD) in a sensitivity analysis. Missing data, including incomplete Bayley-4 scores, scores outside time window and those missing due to LTFU or withdrawal will be imputed (Amelia II R package) using prematurity strata and multiple birth as predictors.^46^ A separate estimand will be defined for the primary outcome excluding participants with all-cause missing data.

##### Secondary analysis

Standardised cognitive scores will also be compared between the two intervention groups using linear mixed effects regression, with partial clustering for multiple births, adjusted for socioeconomic status, parental risk index, sex, birth weight z-score, prematurity strata, antenatal and postnatal steroid use, multiple birth, key neonatal morbidity count and site. Missing data will be imputed or substituted as detailed for the primary estimand.

##### Cost-effectiveness analysis

A separate cost-effectiveness analysis will be performed and reported in accordance with the Consolidated Health Economic Evaluation Reporting Standards (CHEERS) reporting guideline.^47^

### Who will be included in analysis

All randomised participants will be included in each analysis.

### Methods in analysis to handle protocol non-adherence and missing data

Compliance with prescribed light and sound levels recorded at each site and timepoint relative to randomisation, will be reported for the whole cohort and separately for night/day for each stratum and each treatment group.

The handling of missing data will be based on the reason for missingness and documented for each estimand in the SAP. In general, we will employ the Amelia II R package for the multiple imputation of multivariate incomplete data, which uses an algorithm that combines bootstrapping and the expectation-maximisation algorithm.^48^

### Monitoring

#### Data Safety and Monitoring Committee

The DSMC comprises five independent members including four neonatologists (Clinical Prof Shripada Rao—Chair, Assoc Prof Jenny Bowen, Assoc Prof Malcolm Battin, Assoc Prof Jean Du Plessis and an independent biostatistician Dr Charley Budgeon. The role of the DSMC was established in a DSMC Charter developed using the Damocles guidelines (available in an online supplemental file), and finalised on 25 July 2022 after recruitment of 244 participants and prior to the first DSMC meeting. The DSMC aims to safeguard the interests of trial participants, monitor the main outcome measures including safety and efficacy, and monitor the overall conduct of the CIRCA DIEM study. Specific roles of the DSMC are to (1) monitor evidence of efficacy and safety of the CIRCA DIEM study interventions; (2) monitor trial conduct; (3) assess the impact and relevance of new external evidence, trial data and trial conduct to provide recommendations about study continuation; (4) maintain the confidentiality of all trial information not in the public domain including non-disclosure of interim results to trial personnel and committees and (5) advise on trial modifications proposed by investigators or sponsors as requested.

#### Interim analyses

Interim analyses for safety and data completeness will be conducted after 25%, 50% and 75% of the target sample cohort are recruited and have reached 36 weeks PMA (timepoint for assessment of neonatal morbidities). A blinded interim analysis will be performed after 25% and 50% of the participants have reached the 2-year endpoint to assess the assumptions in the sample size calculation. The DSMC will make recommendations to the Trial Steering Committee regarding trial continuation after each interim safety and efficacy analysis. The Trial Steering Committee will make the final decisions about trial termination if required.

### Trial monitoring

Trial conduct is continuously reviewed and audited by the Trial Program Manager, the Trial Coordinator, the Trial Project Officer and the Trial Data Manager. Auditing processes include assessment of the accuracy and completeness of screening, enrolment and randomisation, site-reporting of protocol deviations and verification of the completeness and accuracy of data entered into the Trial electronic database, including prospective comparison and auditing of source data with data entered into the database. Missing data and potential inaccuracies in the data will be audited continuously by the Trial Data Manager.

## Discussion

The application of eye masks and ear plugs overnight and the removal of these devices during the day represents a simple, low-cost and potentially globally applicable intervention for preterm infants that may provide benefits for neurodevelopment in infants born prematurely. Importantly, the integral role of circadian rhythms in coordinating cellular, physiological and developmental processes suggests that a positive outcome to this study could translate into health benefits throughout childhood and into later life. The potential for such future health benefits is underlined by consideration of current thinking that disruption to circadian rhythms may be implicated as a major factor contributing to heart disease, obesity, endocrine, mental health, infectious, inflammatory and cancer disorders, among others.

The international multicentre randomised CIRCA DIEM study will be the largest study undertaken to evaluate the effect of a cycled environment to promote early development of circadian rhythms in infants born <32 weeks. It is the only multicentre randomised controlled trial of a combined cycled light and noise intervention, and the first trial powered to assess and consider the effect of cycled light and noise on cognitive outcomes of very preterm and extremely preterm infants. Importantly, the trial incorporates several substudies to investigate the mechanisms that may underlie any potential clinical outcome differences between groups. As such, the CIRCA DIEM trial will provide a comprehensive insight into the potential value of early curation of endogenous circadian rhythms on not only immediate, but also longer-term outcomes including susceptibility to infection in infancy, sleep and maternal mental well-being.

The key limitation for the study is that it is not possible to blind either the carers or the parents or guardians to group assignment, creating a risk of performance bias due to systematic differences in care. The trial design aims to minimise the impact of this limitation through use of blinding the Trial Steering Committee (including the Trial Biostatistician) and all primary outcome assessors to group assignment. A further limitation is the possibility of attrition bias resulting from perceived differences in care resulting from the intervention or influence of lack of equipoise from some carers. The steering committee aims to minimise the risk of attrition bias through ongoing trial education programmes for parents and staff.

If provision of cycled light and noise is proven effective for improving cognitive scores in infants born before 32 weeks gestation without severe adverse effects, this low-cost, easy to implement and maintain intervention could be rapidly implemented as part of routine clinical care and will highlight the potential benefits of developing a circadian care bundle for incorporation into routine neonatal care. The potential benefits of this intervention could translate to millions of premature infants born globally each year and for their families.

## Ethics and dissemination

### Ethics approval

Primary ethics committee:

Child and Adolescent Health Service Human Research Ethics Committee (under National Mutual Acceptance Scheme): RGS0000006620

Additional ethical approvals were obtained from the following ethics committees for study sites that do not accept approvals under the National Mutual Acceptance Scheme:

1. The University of Western Australia: RA/4/20/5107
2. Ramsay Health Care WA/SA Human Research Ethics Committee: 2019/ETH/1907
3. St John of God Health Care Human Research Ethics Committee: 1657
4. Mercy Health Human Research Ethics Committee: 2023-038
5. Northern A Health and Disability Ethics Committee (New Zealand): 2024 AAM 18428Protocol amendments.^26^

The protocol has undergone 12 amendments since commencement of the study, each of which were submitted to and approved by the Child and Adolescent Health Service Human Research Ethics Committee (Perth). Notification of approved amendments are communicated to other relevant governance and ethics committees as relevant, prior to site notification and distribution of updated documentation.

### Consent

#### Informed consent

Informed parental consent to trial participation is required within the first 168 hours (7 days) post partum for the <28 weeks infants and within the first 72 hours post partum for the ≥28 weeks gestation infants.

#### Additional consent provisions for collection and use of participant data and biological specimens

Consent will be sought to collect and use additional information from health records at the recruiting hospital and other health services as required to inform health economic analysis out to 2 years. Consent will also be sought to recontact parents/guardians in relation to any future study with ethics approval that relates to their child’s participation in the CIRCA DIEM study.

Consent forms included reference to five key substudies designed to run in parallel with the main CIRCA DIEM study. Each of the substudies is described in the main protocol, but will be reported separately with separate published trial protocols:

1. In centres where MRI of the brain is routinely obtained at term equivalent as part of clinical care protocols, permission will be sought from parents to allow CIRCA DIEM researchers to access the images for more detailed analysis, and (in one centre) for collection of additional MRI images. This substudy will investigate whether the intervention influences structural development of the neonatal brain.
2. At KEMH and FSH, the CIRCA DIEM Biological substudy aims to understand if infants receiving the intervention successfully develop their circadian rhythm during hospitalisation through understanding circadian patterns in salivary hormone, buccal cell clock gene expression and actigraphy measurements.
3. At KEMH, the CIRCA DIEM Sleep Sub-study will assess whether the intervention has an effect on infant sleep patterns after the intervention is completed and the infant is discharged home from the hospital.
4. At KEMH, parents/guardians will be invited to enrol their infant into the CIRCA DIEM Infection and Immunology Sub-study (ACTRN12623001073695) to understand the potential implications of the intervention for immunological responses and susceptibility to infection.
5. Infants at all centres where testing is available using the ADOS, will be invited to participate in the Social Communication Sub-study if survey responses conducted as part of the main CIRCA DIEM substudy indicate potential delay or problem with social or communication development. This substudy focuses on whether early restoration of circadian rhythms after preterm birth may reduce the likelihood of later autism diagnosis.

Consent will be sought from parents/guardians who enrol their infant in a substudy that collects biological samples from their infant to store those samples in the CIRCA DIEM Biobank for use in future research approved by a Human Ethics Research Committee. Parent consent will also be sought for CIRCA DIEM Biobank researchers to contact their child again when they reach the age of 18 years to provide consent for samples and health/personal data to be retained into the participant’s adulthood.

#### Consent for publication

Specific consent is obtained from parents for inclusion of deidentified data in publications arising from the study. A model consent form is provided as online supplemental material.

### Confidentiality

Participant data are subject to laws pertaining to data protection and privacy. Study data will be collected and entered on an ISO 27001/2 secure platform provided by the Biometrics team at The Kids Research Institute Australia, hosted on The Kids Research Institute Australia infrastructure within the Perth Children’s Hospital site. Identifiable and potentially identifiable data will be tagged to ensure placement of appropriate limitations of access to these data to a restricted group of approved research staff, and deidentification of downloaded data. Data access groups and user level security will be used to ensure site investigators can only access their own data. All data transactions will be recorded and any changes to data will be logged by data, time and operator.

#### Plans for collection, laboratory evaluation and storage of biological specimens for genetic or molecular analysis in this trial/future use

Samples collected from Western Australian participants in the CIRCA DIEM Study who consent to participate in the Infection and Immunology sub-study will form the basis of the CIRCA DIEM biobank (‘The Biobank’). Samples may include saliva, buccal cells, nasal viral and epithelial swabs, epithelial cells, faeces, blood and urine. Parents (and participants when able to give informed consent) will be invited to give their permission for their infant/child’s samples to be retained in the CIRCA DIEM biobank for future research studies relevant to understanding how prematurity impacts later well-being and/or susceptibility to illness.

### Dissemination plan

Results of the CIRCA DIEM Trial will be disseminated widely through presentations at local, national and international conferences, publication in international peer-reviewed journals, and inclusion on the study website. Information about trial findings will also be communicated directly to the parents/guardians of trial participants through the regular study newsletter. The trial investigators will seek opportunities to communicate study results to the lay public through media and social media avenues.

### Trial status

The current trial protocol is version 3.04 dated 21 June 2024. Recruitment commenced at King Edward Memorial Hospital in February 2019. Additional recruiting sites were added over time. At the time of writing, the DSMC had completed the interim safety reviews of the first three timepoints at 25%, 50% and 75% recruitment and the first efficacy analysis and had recommended that the trial continue unchanged after both reviews. Recruitment is anticipated to be completed by late 2025, with completion of primary outcome data collection anticipated by early 2029. The CIRCA DIEM investigators anticipate releasing primary outcome results by mid-2029. Acknowledging the delay between end of recruitment and availability of primary outcome data, the Trial Steering Committee will publish in hospital morbidity and mortality outcomes after completion of the intervention phase: publication of these secondary outcomes at this time will not influence continuation or integrity of ongoing data collection as outcome assessors and data analyses will remain blinded to participant group assignment.

## Supplementary material

10.1136/bmjopen-2025-112665 online supplemental file 1

10.1136/bmjopen-2025-112665 online supplemental file 2

10.1136/bmjopen-2025-112665 online supplemental file 3
