# Supplementary material for: Efficacy of cycled environmental light and noise during initial hospitalisation for improved cognitive outcomes at 2 years in infants born extremely or very preterm: study protocol for the prospective, randomised, open, blinded endpoint controlled multicentre CIRCA DIEM trial
Source: BMJ Open. 2026 Jul 24;16(7):e112665. doi: 10.1136/bmjopen-2025-112665 (PMC13404858; doi:10.1136/bmjopen-2025-112665)
Supplement: online supplemental file 1 [file bmjopen-16-7-s001.pdf]

[Insert primary site logo here]

Government of Western Australia
Child and Adolescent Health Service

The KIDS RESEARCH INSTITUTE AUSTRALIA — THE CIRCA DIEM STUDY

CIRCA DIEM CHILD AND PARENT/GUARDIAN INFORMED CONSENT

**Title**	**C**ognitive **I**mprovement by Early Restoration of Cir**CAD**ian Rhythms in Very Preterm **I**nfants through **E**nvironmental **M**odification
**Short Title**	The CIRCA DIEM Study
**Protocol Number** (researcher to complete)	*[protocol number]*
**Project Sponsor**	The Kids Research Institute Australia
**Coordinating Principal Investigator**	Professor Jane Pillow
**Site Principal Investigator**	*[site PI name]*
**Location**	*[recruiting site]*

Declaration by Parent/Guardian: please tick to indicate your consent to the following:

**Main CIRCA DIEM Study**	
I have read the Participant Information Sheet and I understand the purpose, procedures, and risks, of the CIRCA DIEM study, as described in the participant information sheet.<br>I have had an opportunity to ask questions and I am satisfied with the answers I have received. I understand that I will be given a signed copy of this document to keep.	☐ Yes<br>☐ No
I agree to my child taking part in the CIRCA DIEM study.<br>I understand that I am free to withdraw my child at any time during the research project. Withdrawing from this study will not affect my child's future medical care.	☐ Yes<br>☐ No
I agree to my own participation in the CIRCA DIEM study.<br>I understand that I am free to withdraw myself at any time during the research project. Withdrawing from this study will not affect my future medical care.	☐ Yes<br>☐ No

**General**	
I agree that research data gathered from the results of this study may be published, but that neither myself nor my child will be identified in any published material.	☐ Yes<br>☐ No
I agree to the storage of data taken from myself and my child for the use in future research that is closely related to this research project, even if my child and/or I withdraw from the study.	☐ Yes<br>☐ No
I agree to be contacted again after my child and I complete the final assessment in relation to any future study with ethics approval relating to my child's participation in the CIRCA DIEM study.	☐ Yes<br>☐ No
I agree that researchers may contact me through my email or my mobile phone, including via a mobile phone app to let me know about upcoming appointments and to collect information about our well-being, including completion of online surveys.	☐ Yes<br>☐ No
I agree that if either I or my child are involved in another research study undertaking any of the same assessments as the CIRCA DIEM study that the results of these common	☐ Yes

assessment items can be shared between CIRCA DIEM and the other research study/studies in which I or my child are participants. I understand that limited identifiable information, such as my child's date of birth, will be shared across these research teams so that my child's results can be accurately logged and stored.	☐ No

A sub-study is an 'add-on' study that helps to answer specific questions within a larger research project. There are three sub-studies associated with the main CIRCA DIEM Study, in which you have the choice to take part. Please tick to show whether you consent to the following Sub-Studies:

**MRI Sub-Study**	
I have read and understood the participant information sheet that includes information about the CIRCA DIEM MRI (magnetic resonance imaging) sub-study. I have had an opportunity to ask questions about the MRI sub-study and I am satisfied with the answers I have received.	☐ Yes<br>☐ No
I agree to my child taking part in the CIRCA DIEM MRI sub-study, and that this participation will allow CIRCA DIEM investigators to access any MRI images of my child's brain that are obtained as part of routine clinical assessment for further analysis and reporting as an outcome of the CIRCA DIEM study.	☐ Yes<br>☐ No

**Sleep Sub-Study**	
I have read and understood the participant information sheet that includes information about the CIRCA DIEM Sleep sub-study.<br>I have had an opportunity to ask questions about the Sleep sub-study and I am satisfied with the answers I have received.	☐ Yes<br>☐ No
I agree to complete surveys about my child's sleep prior to my child's discharge from hospital and again at 2 months and 6 months of age (corrected).	☐ Yes<br>☐ No
I agree to complete a sleep diary about my own sleep prior to my child's discharge from hospital and again when my child is 2 months and 6 months of age (corrected).	☐ Yes<br>☐ No
Prior to my child's discharge from hospital and again at 2 months and 6 months of age (corrected), I agree to the collection of:<br>1) some saliva from cotton swabs placed in my child's mouth in the morning and in the evening to measure the 'sleep hormone" called melatonin.<br>2) information about my child's sleep behaviours from a watch-like device that my child will wear on their leg<br>3) information about my own sleep behaviours from a watch-like device that I will wear on my wrist.	☐ Yes<br>☐ No<br>☐ Yes<br>☐ No<br>☐ Yes<br>☐ No

Infection and Immunology Sub-Study	
I have read and understood the participant information sheet that includes information about the CIRCA DIEM Infection and Immunology sub-study.<br><br>I have had an opportunity to ask questions about the Infection and Immunology sub-study and I am satisfied with the answers I have received.	☐ Yes ☐ No
I agree to my child's participation in the CIRCA DIEM Infection and Immunology sub-study including ongoing follow-up through a mobile app and surveys that will track my infant's temperatures, infections, GP visits and hospitalisations during the first 2 years of life and what treatment they receive.	☐ Yes ☐ No
I understand that my agreement will allow CIRCA DIEM investigators to access medical and hospital records for information about infections that my infant may develop over the first two years of life.	☐ Yes ☐ No
I understand that my placenta was sent to pathology when I gave birth to my preterm baby and agree to the retention of a small portion of my placenta for further analysis as part of the CIRCA DIEM Infection and Immunology sub-study.	☐ Yes ☐ No
I agree to the collection and testing of the following samples from my baby. I understand that these samples may be collected<br>⇒ around the time of enrolment of my baby into the study<br>⇒ around the time of my child's discharge from hospital<br>⇒ around the time of my child's follow-up appointment at 1 years of age	
1) a swab from inside the nose (similar to a RAT test for COVID-19) to collect nasal fluid	☐ Yes ☐ No
2) a brushing from the nose to collect nasal cells prior to discharge and at the 1 year follow up appointment	☐ Yes ☐ No
3) collection of some cells from the inside of my baby's cheek	☐ Yes ☐ No
4) collection of samples of my baby's stools (poo) from their nappy	☐ Yes ☐ No
5)	
6) collection of 3-4 drops of blood (0.3 millilitres) at study entry and prior to discharge	☐ Yes ☐ No
7) collection of 5 mL of blood at the 1 year follow-up assessment	☐ Yes ☐ No
I agree for my infant/child to have a nasal swab to determine if they have a bacterial or viral infection if my infant/child has symptoms of respiratory infections and temperatures of 38.5 degrees Celsius or more for three days.<br><br>I understand that a research nurse may come to visit my home to collect this nasal swab from my child.	☐ Yes ☐ No

CIRCA DIEM Biobank	
I have read and understood the participant information sheet that includes information about the CIRCA DIEM Biobank. I have had an opportunity to ask questions about the Biobank and I am satisfied with the answers I have received.	☐ Yes ☐ No
I agree to donate my child's sample(s) to the CIRCA DIEM Biobank. I agree that my child's sample can be used by ethically approved research studies across Australia and that I will not be told what research my child's sample(s) is used for.<br><br>I understand that my child will be approached for consent if their sample(s) is to be retained after they turn 18 years old.	☐ Yes ☐ No
I agree to donate the placenta sample belonging to me and my child to the CIRCA DIEM Biobank. I agree that our placenta sample can be used by ethically approved research studies across Australia and that I will not be told what our placenta sample is used for.	☐ Yes ☐ No

[Insert primary site logo here]

Government of Western Australia
Child and Adolescent Health Service

INFORMED CONSENT FOR CHILD PARTICIPATION IN CIRCA DIEM

Name of Child: ______________________________

Name of Parent/Guardian: ______________________________

Signature of Parent/Guardian: ______________________________ Date: ____/____/____

INFORMED CONSENT FOR PARENT/GUARDIAN PARTICIPATION IN CIRCA DIEM

Name of Parent/Guardian: ______________________________

Signature of Parent/Guardian: ______________________________ Date: ____/____/____

Declaration by Study Principal Investigator

I have given a verbal explanation of the research project; its procedures and risks and I believe that the parent/guardian has understood that explanation.

Name of Recruiting Principal Investigator or Research Team Member: ______________________________

Signature of Recruiting Principal Investigator or Research Team Member: ______________________________ Date: ____/____/____

This study has approval from the **Child and Adolescent Health Service Human Research Ethics Committee** based in Western Australia.
