# Supplementary material for: Efficacy of cycled environmental light and noise during initial hospitalisation for improved cognitive outcomes at 2 years in infants born extremely or very preterm: study protocol for the prospective, randomised, open, blinded endpoint controlled multicentre CIRCA DIEM trial
Source: BMJ Open. 2026 Jul 24;16(7):e112665. doi: 10.1136/bmjopen-2025-112665 (PMC13404858; doi:10.1136/bmjopen-2025-112665)
Supplement: online supplemental file 2 [file bmjopen-16-7-s002.pdf]

Data and Safety Monitoring Committee Charter for the CIRCA DIEM STUDY

This document has been developed using the DAMOCLES guidelines[1]

1 FULL TITLE OF THE RANDOMISED TRIAL:

COGNITIVE **I**MPROVEMENT BY EARLY RESTORATION OF CIR**CAD**IAN RHYTHMS IN VERY PRETERM **I**NFANTS THROUGH **E**NVIRONMENTAL **M**ODIFICATION

2 ABBREVIATED STUDY TITLE

The CIRCA DIEM study

3 VERSION:

v1.03, Dated: 30 July 2024, appropriate for protocol v3.04 (approved 21 June, 2024) and later.

4 LEAD HUMAN RESEARCH ETHICS COMMITTEE (HREC):

Child and Adolescent Health Service, Western Australia

5 HREC REFERENCE NUMBER:

RGS0000000954 and RGS0000006620

6 TRIAL SPONSOR

The Kids Research Institute Australia (formerly known as The Telethon Kids Institute)

7 ANZCTR TRIAL ID:

ACTRN12618000371291 (CIRCA DIEM Main Study)

ACTRN12623001073695 (CIRCA DIEM Infection and Immunology Substudy)

8 SUMMARY OF THE STUDY PROTOCOL:

8.1 Background:

The CIRCA DIEM study is a multicentre, prospective, blinded end-point (PROBE) parallel controlled trial, which compares long term neuro-developmental outcomes of premature babies exposed to cycled light and sound during hospitalisation, versus those who receive routine care.

8.2 Aim:

The overall aim of the CIRCA DIEM study is to establish whether targeted individual diurnal cycling of environmental light and noise levels results in improved neurodevelopmental social, psychological, physiological, and health economic outcomes of very preterm infants compared to more constant background lighting and noise environments.

8.3 Hypothesis:

8.3.1 Primary hypothesis

Exposure to cycled light and noise from birth until discharge home will increase cognitive score on the Bayley Scales of Infant Development 4th Edition (Bayley-4) at 2 years corrected age.

8.3.2 Secondary hypotheses

Exposure to cycled light and noise from birth until discharge home will: 1) improve neurodevelopmental sub-scores and neurobehaviour at 2 years corrected age; 2) not increase mortality or neonatal morbidities; 3) result in more rapid development of endogenous circadian rhythms; 4) reduce parental depression and anxiety and improve parent-infant interactions; and 5) decrease socio-economic costs of premature birth.

8.4 Outcomes:

8.4.1 Primary outcome:

Mean difference in cognitive score on Bayley Scales of Infant Development (BSID)-4 at two years corrected age.

8.4.2 Secondary outcomes:

Comprise short-term and longer-term neurodevelopmental and clinical outcomes as well as targeting physiological and molecular indicators of circadian rhythm development to inform mechanism and effectiveness of the intervention. Economic evaluation of the intervention will also be undertaken.

8.5 Intervention:

Infants assigned to the intervention group will be exposed to a minimum of 300 lux and a maximum of 600 lux during the day and will not receive daytime protection from noise unless receiving high-frequency oscillatory ventilation. During the evening, the intervention group will wear black-out eye masks and silicone ear plugs positioned over the external pinna of the ears, and kept in position by the band of the eye mask. The day cycle (300-600 lux and no noise

protection) will extend from 6 am to 8 pm (14 hours). The nocturnal cycle (eye masks and ear plugs) will be applied from 8 pm to 6 am (10 hours).

Standardised eye masks, and silicone plugs will be supplied, and applied during each period by the nurse caring for the infant.

The control group will receive routine care with no cycled light or noise (daytime-night-time lux difference < 100 lux).

Both groups will have standardised timing of caffeine medication (or other neurostimulants) to avoid confounding of circadian rhythms by inappropriate medication timing.

8.6 Sample size:

A sample size of 954 is required to detect a difference of at least 4 points on the cognitive sub-score at 2 years corrected postnatal age. This calculated sample size assumes 90 % power, a 5 % significance level, standard deviation of 15 points, a 2-sided t-test, 10 % loss to follow-up, and up to 30 % of eligible participants from multiple births.

8.7 The CIRCA DIEM study investigators:

8.7.1 Principal investigator (PI):

Prof Jane Pillow (JP)

8.7.2 Co-Chief Investigators:

Prof Rod Hunt (RH)
Prof Peter Anderson (PA)
Dr Peter Mark (PM)
Prof Alicia Spittle (AS)
Prof Andrew Whitehouse (AW)
Dr Julie Marsh (JM)
Prof Catherine Elliott (CE)
Prof Nadia Badawi (NB)

8.7.3 Associate Investigators (AIs):

Mrs Catherine Gwynn (CG) – Consumer representative
Prof Elizabeth Geelhoed (EG) – Health Economist
Clin A/Prof Mary Sharp (MS) -Site PI, KEMH
Dr Calum Roberts (CR) – Site PI, Monash Newborn
Dr Daphne D’Cruz (DD) – Site PI, Westmead
Clin Prof Adrienne Gordon (AG) – Site PI, Royal Prince Alfred Hospital
Assoc/Prof Michael Stark (MS)– Site PI, Women’s & Children’s Hospital
Prof Helen Liley (HL) – Site PI, Mater Mother’s Hospital

9 THE ROLE AND OPERATION OF THE DSMC

9.1 Purpose of the DSMC document:

To describe the membership, terms of reference, process, roles and responsibilities, authority, decision-making and relationships with other trial personnel and committees of the independent Data and Safety Monitoring Committee (DSMC) for the CIRCA DIEM study. This document includes the timing of meetings, methods of providing information to and from the DSMC, frequency and format of meetings. The background to this trial and the trial objectives are described in the protocol.

9.2 Aim of the DSMC:

To safeguard the interests of trial participants, monitor the main outcome measures including safety and efficacy, and monitor the overall conduct of the CIRCA DIEM study.

9.3 Specific role of DSMC:

1. To monitor evidence of efficacy and safety of the CIRCA DIEM study interventions by
   a. reviewing the reports of the serious adverse events provided by the principal investigator
   b. reviewing the reports from the regular conduct of interim analysis of the data.
2. To monitor trial conduct by reviewing the reports of trial progress, including but not limited to screening, enrolment lost to follow-up, protocol violation and adherence rates, provided by the principal investigator.
3. To assess the impact and relevance of new external evidence, trial data (accrual, safety & efficacy) and trial conduct to provide recommendations regarding the continuation of the study.
4. To maintain the confidentiality of all trial information that is not in the public domain, including non-disclosure of interim results to trial personnel or committees.
5. To advise on trial modifications proposed by investigators or sponsors as requested, e.g. to inclusion criteria, trial endpoints, etc.

9.4 Important safety related outcomes that will be monitored by the DSMC:

Composite outcome of mortality before discharge OR severe intraventricular haemorrhage (grade 3 or 4 IVH), OR necrotising enterocolitis (stage IIA or higher) OR retinopathy of prematurity (ROP) requiring treatment. In addition, if there are excess of individual adverse outcomes, they will be carefully reviewed. Any other serious outcome reported by the PI will be considered by the DSMC.

9.5 Membership:

The DSMC is a multidisciplinary group that have collective experience/expertise in the management of participants with condition(s) relevant to the study, anticipated adverse events, and in the conduct and monitoring of randomised clinical trials. The committee also includes members with recognised biostatistical expertise. The members should be independent of the trial, which is defined as individuals who are (i) not named as a CIRCA DIEM investigator or site principal and (ii) not employed by the trial or the Sponsor and (iv) not named as an author on

any publications arising from the trial and (v) have no vested interests in the interventions used in the trial. The Chair will be nominated and agreed by the Trial Steering Committee (TSC). The Chair is expected to facilitate and summarise discussions.

1. Clin A/Prof Shripada Rao (SR): Neonatologist, Perth Children's Hospital; DSMC Chair.
2. A/Prof Malcom Battin (MB): Neonatologist, Starship Hospital, Auckland, New Zealand.
3. A/Prof Jenny Bowen (JB): Neonatologist, Royal North Shore Hospital, NSW.
4. A/Prof Jean Du Plessis (JDP): Neonatologist, Fiona Stanley Hospital, WA.
5. Dr Charley Budgeon (CB): Biostatistician, The University of Western Australia, WA.

9.6 Potential Competing Interests

Any competing interests, both real or potential, should be disclosed. These are not restricted to financial matters – involvement in other trials or intellectual investment could be relevant. Although members may well be able to act objectively despite such connections, complete disclosure enhances credibility. DSMC members should disclose to the Chairperson any other conflicts they consider relevant. Any members who develop significant conflicts of interest during the course of the trial should inform the Chair and may need to consider resigning from the DSMC. The membership of the DSMC will be reviewed every year. Trial independence is defined above under Membership.

9.7 Payments

If required, reasonable reimbursement for time, and any travel or accommodation costs will be provided. No other payments or rewards will be given to members.

9.8 Production of reports for the DSMC:

The Trial Management Group (TMG) will produce open interim reports (i.e. aggregated over all study participants and blinded) to the DSMC, which will contain details on the trial progress, adherence to intervention, data quality and overall safety.

The trial statistician (or nominated delegate) will produce closed interim reports (i.e. summarised by masked treatment allocation) that summarise safety data and the primary efficacy endpoint. The format of the closed interim reports will be documented in the statistical analysis plans and contain a minimum of:

1. Consort style flowchart of eligible participants at follow-up milestones (visits) by masked intervention allocations.
2. List of protocol violations.
3. Primary efficacy outcome summarised by masked intervention allocation.
4. Summary of adverse events, coded by MedDRA preferred term, by masked intervention allocation.

Masking will be used so the DSMC will not know to which group the individual study infants belong. All reports produced for the DSMC will be confidential and restricted to the members of the DSMC.

Timing of reports for review by the DSMC

Where possible, the DSMC should receive data/reports (prepared by the TMG and/or trial statistician) at least 1 week prior to meeting. The DSMC members should delete or destroy or store securely the papers and files after each meeting. After the trial is reported, the DSMC Chairperson should send a copy of all minutes from open sessions to the TMG, and minutes from closed sessions to the trial statistician or nominated delegate. Reports and minutes will be stored and archived securely by the TMG and trial statistician, respectively. DSMC members should then destroy all interim reports and minutes.

9.9 Responsibility of PI:

PI or delegate will provide data to the DSMC for interim reports and be available to attend meetings as necessary.

9.10 Scheduling, Quorum and Organisation of DSMC meetings:

The Chairperson is responsible for facilitating the meetings, summarising discussions and communicating recommendations to the chair of the TSC. A facilitator (from the TMG) will be responsible for the organisation of meetings and should be copied into all communications with and between the DSMC.

The meetings will be organised periodically for approximately one week after the expected production of the interim report after the first 25 %, 50 % and 75 % of enrolled CIRCA DIEM participants have been discharged from hospital or reached term equivalent gestation. Meetings can be convened at the request of the TMG, Chief Investigators, or any DSMC member. Dates for DSMC meetings should be agreed in advance, where possible, but may need to be altered subject to the timing of the interim analyses. The meetings will be through electronic communications. Videoconference will be undertaken if necessary. The DSMC will invite the PI and the trial statistician (or nominated delegate) for meetings as necessary. A mixture of open and closed sessions will be held, and if necessary executive sessions (see section below). A meeting of the DSMC is considered quorate if the Chairperson (unless otherwise agreed), one DSMC statistician, and at least one other DSMC member is present.

Role of open, closed and executive sessions

DSMC meetings may consist of up to three sessions: open, closed and executive. The coordinating chief investigators, co-investigators, trial statistician (or nominated delegate) and trial management group (TMG) may attend the open session. The Co-ordinating CIs should be invited and should be available to attend open sessions of the DSMC meeting. The co-investigators, trial statisticians and TMG members will not usually be expected to attend but can attend open sessions when necessary. The purpose of the open session is to provide relevant information to the DSMC about general aspects of the trial.

Only members of the DSMC and trial statistician may attend and participate in the closed session of the meetings to guide the DSMC through the unblinded interim efficacy report, respond to questions and clarifications and to take notes about further unblinded summaries or analyses requested by the DSMC. During the closed session, the Chairperson conducts the review of all issues, seeks consensus for recommendations, and if necessary, puts each issue to a vote.

An executive session of the meeting may be held that is restricted to DSMC members only. During these sessions, the DSMC may discuss any unblinded analyses and any sensitive issues surrounding the clinical trial under review. At the close of the meeting, the DSMC Chairperson is responsible for communicating the recommendations to the Chair of the TSC.

9.11 DSMC recommendations and reporting

The DSMC will report its recommendations in writing to the Chairperson of the TSC, usually within 2 weeks of the meeting. The reporting will be in the form of electronic mail from the chair of the DSMC to the study investigators, and carbon copied to other members of the DSMC**.** If the trial is to continue largely unchanged, it is useful for the report from the DSMC to include a summary paragraph suitable for trial promotion purposes i.e. to be circulated to trial sites**, maintaining the blinded status of the trial results as appropriate.**

Recommendations may be, but are not restricted to:

- The study continues as planned.
- The study can continue with proposed modification in the protocol (the modifications may need HREC approval).
- Early stopping for superiority of a new treatment requires proof beyond a reasonable doubt. Hence the DSMC will use p value of <0.001 if the intervention arm shows better primary outcomes compared to controls (Haybittle-Peto criteria) on interim analyses.[2,3] It is known that RCTs stopped early for benefit often show implausibly large treatment effects, particularly when the number of events is small.[4] Hence the DSMC's base-case approach would be to ensure the completion of full sample size recruitment.
- The recommendation of early stopping of the trial for safety: The stopping boundary needs to be less stringent for this purpose;[3] hence the DSMC will use a p value of<0.01 if the intervention arm shows worse safety outcomes compared to controls.[3]
- P values will not be the sole factor in making decisions by the DSMC. The collective opinion of the members of the DSMC will be an important factor while making such decisions.

9.12 How decisions are reached within the DSMC:

Efforts will be made by the DSMC to reach a unanimous decision. If the DSMC cannot achieve a unanimous decision, a vote may be taken.

9.13 Meeting Minutes

Separate records will be required for open and closed sessions with minutes made by the appropriate attending member of the TSC/TMG and DSMC, respectively. This will usually be the Trial Manager for the open session and the Chairperson or Trial statistician for the closed session. The DSMC Chairperson should approve in writing or by email any minutes or notes.

9.14 Attendance

Efforts should be made to ensure that all members can attend. For members that are unable to attend the meeting, minutes will be circulated and any queries will be addressed by the Chairperson. All members will be sent a reminder email with agenda and approved minutes from previous meeting. Meetings will continue as long as at least a quorum is achieved (see above) and any recommendations for actions required will be noted in the minutes. If the DSMC is

considering recommending major action after such a meeting, the DSMC Chairperson should communicate with the absent members as soon after the meeting as possible to check they agree. If there is any disagreement, a further meeting should be arranged with the full DSMC.

The DSMC should be available to provide independent advice as required, not just when meetings are scheduled. Major trial issues may need to be dealt with between meetings, by phone or by email. The DSMC should maintain confidentiality of all information it receives. Members should not discuss confidential issues from their involvement in the study until trial results have been published.

9.15 How decisions or recommendations will be reached within the DSMC

The Chairperson is to summarise discussions and encourage consensus; it is usually best for the Chairperson to give their own opinion last. Every effort should be made for the DSMC to reach a unanimous decision. If the DSMC cannot achieve this, a vote may be taken, although details of the vote should not be routinely included in the report to the TSC as these may inappropriately convey information about the state of the trial data. It is important that the implications (e.g. ethical, statistical, practical, financial) for the trial be considered before any recommendation is made.

9.16 Relationship with Trial Steering Committee (TSC)

The TSC is the oversight and decision-making body and is delegated this role by the sponsor. The DSMC is advisory to the TSC. The TMG, and DSMC make comments, requests and recommendations to the TSC. If the DSMC has serious problems or concerns with a TSC decision, a joint meeting of these trial committees should be held. The information to be shown would depend upon the action proposed and the DSMC's concerns. Depending on the reason for the disagreement, confidential data may need to be revealed to all those attending such a meeting. The meeting would be Chaired by a senior member of the sponsor staff or an external, independent expert who is not directly involved with the trial.

9.17 Acknowledgements:

The trial results will be published in a correct and timely manner. The process of publication should be overseen by the TSC. The DSMC members will be provided the draft of the manuscript for comments prior to submission to a peer reviewed journal or for conference purposes. The DSMC members will be acknowledged in the study publication. A brief summary of the timings and conclusions of DSMC meetings will be included in the body of this paper.

10 References:

1. DAMOCLES Study Group, NHS Health Technology Assessment Programme. A proposed charter for clinical trial data monitoring committees: helping them to do their job well. *Lancet* 2005 Feb 19-25;365(9460):711-22.
2. Schulz KF, Grimes DA. Multiplicity in randomised trials II: subgroup and interim analyses. *Lancet* 2005;365:1657-61.
3. Pocock SJ, Clayton TC, Stone GW. Part 4 of a 4-part series on statistics for clinical trials. *J Am Coll Cardiol.* 2015;66(25):2886-98
4. Montori VM, Devereaux PJ, Adhikari NK, Burns KE, et al. Randomized trials stopped early for benefit: a systematic review. *JAMA*. 2005 Nov 2;294(17):2203-9. doi: 10.1001/jama.294.17.2203. PMID: 16264162.

11 AGREEMENTS

DSMC members formally acknowledge their agreement to join the group by signing this document indicating that they:

1. agree to be a member;
2. agree with the contents of this charter;
3. and have declared any potential conflicts of interest.

Shripada Rao (Aug 14, 2024 11:53 GMT+8) — 14/08/24

Clin A/Prof Shripada Rao — *Date*

Potential conflicts of interest: None

15/08/24

A/Prof Malcolm Battin — *Date*

Potential conflicts of interest: None

Jenny Bowen (Aug 16, 2024 14:34 GMT+10) — 16/08/24

A/Prof Jenny Bowen — *Date*

Potential conflicts of interest: None

Jean Du Plessis (Aug 14, 2024 07:23 GMT+2) — 14/08/24

A/Prof Jean Du Plessis — *Date*

Potential conflicts of interest: None

15/08/24

Dr Charley Budgeon — *Date*

Potential conflicts of interest: None

DSMC Charter CIRCA DIEM V1.03

Final Audit Report 2024-08-16

Created:	2024-08-14
By:	Gillian Phillipson (Gillian.Phillipson@telethonkids.org.au)
Status:	Signed
Transaction ID:	CBJCHBCAABAANtirOF0rbyW6YiOrYw1TAkyZTPE_lUKc

"DSMC Charter CIRCA DIEM V1.03" History

Document created by Gillian Phillipson (Gillian.Phillipson@telethonkids.org.au)
2024-08-14 - 2:56:16 AM GMT

Document emailed to Shripada Rao (shripada.rao@health.wa.gov.au) for signature
2024-08-14 - 2:56:22 AM GMT

Document emailed to Malcolm Battin (malcolmb@adhb.govt.nz) for signature
2024-08-14 - 2:56:22 AM GMT

Document emailed to Jenny Bowen (jenny.bowen@health.nsw.gov.au) for signature
2024-08-14 - 2:56:23 AM GMT

Document emailed to Jean Du Plessis (jean.duplessis@health.wa.gov.au) for signature
2024-08-14 - 2:56:23 AM GMT

Document emailed to Charley Budgeon (charley.budgeon@uwa.edu.au) for signature
2024-08-14 - 2:56:23 AM GMT

Email viewed by Jenny Bowen (jenny.bowen@health.nsw.gov.au)
2024-08-14 - 2:56:42 AM GMT

Email viewed by Shripada Rao (shripada.rao@health.wa.gov.au)
2024-08-14 - 3:52:32 AM GMT

Document e-signed by Shripada Rao (shripada.rao@health.wa.gov.au)
Signature Date: 2024-08-14 - 3:53:42 AM GMT - Time Source: server

Email viewed by Jean Du Plessis (jean.duplessis@health.wa.gov.au)
2024-08-14 - 5:21:41 AM GMT

Document e-signed by Jean Du Plessis (jean.duplessis@health.wa.gov.au)
Signature Date: 2024-08-14 - 5:23:17 AM GMT - Time Source: server

Email viewed by Malcolm Battin (malcolmb@adhb.govt.nz)

2024-08-14 - 9:19:00 PM GMT

Document e-signed by Malcolm Battin (malcolmb@adhb.govt.nz)

Signature Date: 2024-08-14 - 9:22:18 PM GMT - Time Source: server

Email viewed by Charley Budgeon (charley.budgeon@uwa.edu.au)

2024-08-15 - 9:09:06 AM GMT

Document e-signed by Charley Budgeon (charley.budgeon@uwa.edu.au)

Signature Date: 2024-08-15 - 9:10:25 AM GMT - Time Source: server

Document e-signed by Jenny Bowen (jenny.bowen@health.nsw.gov.au)

Signature Date: 2024-08-16 - 4:34:15 AM GMT - Time Source: server

Agreement completed.

2024-08-16 - 4:34:15 AM GMT
