# Supplementary material for: Efficacy of cycled environmental light and noise during initial hospitalisation for improved cognitive outcomes at 2 years in infants born extremely or very preterm: study protocol for the prospective, randomised, open, blinded endpoint controlled multicentre CIRCA DIEM trial
Source: BMJ Open. 2026 Jul 24;16(7):e112665. doi: 10.1136/bmjopen-2025-112665 (PMC13404858; doi:10.1136/bmjopen-2025-112665)
Supplement: online supplemental file 3 [file bmjopen-16-7-s003.docx]

**Table S1: Schedule of Assessments**

| **Timepoint** | **Enrolment**  (Strata 1:<168h;  Strata 2: <72h) | **Randomisation** (within 24h of enrolment) | **d7** | **d14** | **d28** | **d56** | **36w PMA** | **40w PMA** | **44w PMA** | **2m cPNA** | **3m cPNA** | **6m cPNA** | **12m cPNA** | **24 m cPNA** |
| --- | --- | --- | --- | --- | --- | --- | --- | --- | --- | --- | --- | --- | --- | --- |
| **Enrolment** |  |  |  |  |  |  |  |  |  |  |  |  |  |  |
| Eligibility screen | X |  |  |  |  |  |  |  |  |  |  |  |  |  |
| Informed consent | X |  |  |  |  |  |  |  |  |  |  |  |  |  |
| Randomisation data |  | X |  |  |  |  |  |  |  |  |  |  |  |  |
| Baseline infant data |  | X |  |  |  |  |  |  |  |  |  |  |  |  |
| **Intervention** |  |  |  |  |  |  |  |  |  |  |  |  |  |  |
| Continuous |  | X | X | X | X | X | X | X | X |  |  |  |  |  |
| **Outcome assessments** |  |  |  |  |  |  |  |  |  |  |  |  |  |  |
| Monitoring of intervention application |  |  | X | X | X | X | X | X | X |  |  |  |  |  |
| In hospital safety data |  |  | X | X | X | X | X | X | X |  |  |  |  |  |
| Growth |  |  | X | X | X | X | X | X | X | X |  | X | X | X |
| Neonatal morbidity |  |  |  |  |  |  | X | X | X |  |  |  |  |  |
| Completion of hospital data |  |  |  |  |  |  |  |  | X |  |  |  |  |  |
| Longer term outcome surveys |  |  |  |  |  |  |  |  |  | X |  | X | X | X |
| General movements assessment |  |  |  |  |  |  |  |  |  |  | X |  |  |  |
| Bayley-4 assessment |  |  |  |  |  |  |  |  |  |  |  |  |  | X |
| **Maternal assessments** |  |  |  |  |  |  |  |  |  |  |  |  |  |  |
| Maternal demographic & pregnancy data | X |  |  |  |  |  |  |  |  |  |  |  |  |  |
| Shift work survey |  |  | X |  |  |  |  |  |  | X |  |  |  | X |
| Mental wellbeing |  |  | X |  |  |  |  |  |  | X |  | X | X | X |

PMA, postmenstrual age; cPNA, corrected postnatal age

| Table S2: List of Abbreviations | |
| --- | --- |
| AAP | American Academy of Pediatrics |
| BPD | Bronchopulmonary dysplasia |
| cPNA | Corrected postnatal age |
| CSBS-ITC | Communication and Symbolic Behaviour Scales Infant-Toddler Checklist |
| ICROP3 | International Classification of Retinopathy of Prematurity (3^rd^ Edition) |
| ITSEA | Infant Toddler Social-Emotional Assessment |
| IVH | Intraventricular haemorrhage |
| MDI | Mental develop |
| NEC | Necrotising enterocolitis |
| NICU | Neonatal intensive care units |
| NIDCAP | Neonatal Individualised Developmental Care and Assessment Program |
| PCF | Participant consent form |
| PMA | Postmenstrual age |
| PVL | Periventricular leukomalacia |
| REDCap | Research Electronic Database Capture |
| ROP | Retinopathy of prematurity |
| SD | Standard deviation |
| GAD-7 | Generalised Anxiety Disorder- 7 |
| EPDS | Edinburgh Postnatal Depression Scale |
| SAP | Statistical Analysis Plan |
| CRF | Clinical record form |
| DSMC | Data Safety Monitoring Committee |
| AE | Adverse event |
| SAE | Serious adverse event |
