# Supplementary material for: Efficacy of cycled environmental light and noise during initial hospitalisation for improved cognitive outcomes at 2 years in infants born extremely or very preterm: study protocol for the prospective, randomised, open, blinded endpoint controlled multicentre CIRCA DIEM trial
Source: BMJ Open. 2026 Jul 24;16(7):e112665. doi: 10.1136/bmjopen-2025-112665 (PMC13404858; doi:10.1136/bmjopen-2025-112665)
Supplement: Supplementary file 4 [file bmjopen-2025-112665.reviewer_comments.pdf]

PEER REVIEW HISTORY

BMJ Open publishes all reviews undertaken for accepted manuscripts. Reviewers are asked to complete a checklist review form and are provided with free text boxes to elaborate on their assessment. These free text comments are reproduced below.

ARTICLE DETAILS

Title (Provisional)

Efficacy of cycled environmental light and noise during initial hospitalisation for improved cognitive outcomes at 2 years in infants born extremely or very preterm: Study protocol for the prospective, randomised, open, blinded endpoint controlled multicentre CIRCA DIEM trial

Authors

Pillow, J Jane; Hunt, Rod W; Marsh, Julie A.; Anderson, Peter J.; Mark, Peter J.; Spittle, Alicia J; Whitehouse, Andrew J.; Badawi, Nadia; CIRCA DIEM Study, The

---

VERSION 1 - REVIEW

**Reviewer**	**1**
**Name**	**Patel, Archana**
**Affiliation**	**Seattle Children's Hospital, Neurology**
**Date**	**16-Jan-2026**
**COI**	**None**

Dear authors, this study sounds interesting, impactful, and is well described. Only feedback is use (if available) of questionnaires or other measures to assess consolidated sleep/quality of life around sleep metrics for caregivers in long-term outcomes.

**Reviewer**	**2**
**Name**	**Tas Arslan , Fatma**
**Affiliation**	**Selçuk Üniversitesi**
**Date**	**04-Mar-2026**
**COI**	**None**

Dear author, I believe that the results of your study protocol titled "Study Scientific title Cognitive Improvement at 2 years corrected postnatal age through early Restoration of cirCADian rhythms in very preterm infants via environmental factors" will shed light on significant improvements in newborn health

VERSION 1 - AUTHOR RESPONSE

We note the suggestion of Reviewer 1 regarding inclusion of questionnaires or other measures to assess consolidated sleep/quality of life around sleep metrics for caregivers in long-term outcomes. A Sleep Substudy is referred to in the Consent (32) section but will be presented separately as we aimed to keep this protocol manuscript confined to detail the core parent study and its key short- and long-term outcomes.

VERSION 2 - REVIEW

**Reviewer**	**1**
**Name**	**Patel, Archana**
**Affiliation**	**Seattle Children's Hospital, Neurology**
**Date**	**11-May-2026**
**COI**	

Thank you for the minor revisions; all concerns for my review have been addressed.
